# Cross-Linked Polyvinylimidazole Complexed with Heteropolyacid Clusters for Deep Oxidative Desulfurization

**DOI:** 10.3390/molecules29174238

**Published:** 2024-09-06

**Authors:** Zhuoyi Ren, Jiangfen Sheng, Qibin Yuan, Yizhen Su, Linhua Zhu, Chunyan Dai, Honglei Zhao

**Affiliations:** 1 Engineering Research Center of Tropical Marine Functional Polymer Materials of Hainan Province, Key Laboratory of Water Pollution Treatment and Resource Reuse of Hainan Province, Key Laboratory of Functional Organic Polymers of Haikou, Hainan Normal University, Haikou 571158, China; 15084696781@163.com (Z.R.); 202212070300004@hainnu.edu.cn (Q.Y.); 202212070300017@hainnu.edu.cn (Y.S.); 2 Jiangsu Jitri Carbon Fiber & Composite Application Technologies Research Institute Co., Ltd., Changzhou 213000, China; shengjiangfeng@cfct-jitri.com; 3 Hainan Lesso Technology Industrial Co., Ltd., Dingan 571200, China

**Keywords:** heteropolyacid clusters, polyvinylimidazole, cross-linking, deep oxidative desulfurization

## Abstract

The combustion of fuel with high sulfur concentrations produces a large number of sulfur oxides (SO_x_), which have a range of negative effects on human health and life. The preparation of catalysts with excellent performance in the oxidative desulfurization (ODS) process is highly effective for reducing SO_x_ production. In this paper, cross-linked polyvinylimidazole (VE) was successfully created using a simple ontology aggregation method, after which a catalyst of polyvinylimidazolyl heteropolyacid clusters (VE-HPA) was prepared by adding heteropolyacid clusters. Polyvinylimidazolyl-phosphotungstic acid (VE-HPW) showed an outstanding desulfurization performance, and the desulfurization efficiency reached 99.68% in 60 min at 50 °C with H_2_O_2_ as an oxidant. Additionally, the catalyst exhibited recyclability nine consecutive times and remained stable, with a removal rate of 98.60%. The reaction mechanism was eventually proposed with the assistance of the free radical capture experiment and GC-MS analysis.

## 1. Introduction

The combustion of large quantities of fossil fuels significantly impacts the environment, highlighting the urgent need for deep desulfurization of these fuels. In recent years, many countries have gradually required the sulfur content in diesel fuel to be less than 10 ppm [1]. The traditional method of industrial desulfurization is hydrodesulfurization (HDS), but it requires severe conditions of high temperature and high pressure in the process [2,3,4,5]. HDS also produces toxic H_2_S gas and by-products. Non-hydrodesulfurization technologies such as extractive desulfurization (EDS) [6,7,8], adsorption desulfurization (ADS) [9,10,11], biological desulfurization (BDS) [12,13], and oxidative desulfurization (ODS) do not require the participation of hydrogen [14,15], which has emerged as a critical focal point in the realm of desulfurization research.

With thorough research on deep desulfurization, ODS has been favored by more scientific researchers [16,17,18,19]. The principle of oxidative desulfurization (ODS) involves the oxidation of sulfides into more polar sulfones through the combined action of oxidants and catalysts. ODS is a simple, safe, and environmentally friendly process, making it one of the most promising technologies for effectively removing thiophene-based organosulfur compounds with significant steric hindrance under moderate conditions, thereby producing ultraclean fuels [20,21]. He et al. used Mo-doped W_18_O_49_ nanowire clusters for oxidative desulfurization, which showed that the removal efficiency of DBT could reach 95.7% at 60 °C in 60 min [22]. Zhu et al. synthesized a series of MoO_2_-supported CNT catalysts and achieved a desulfurization efficiency of 99% for DBT, 4-MDBT, and 4,6-DMDBT in 40 min [23].

Polyoxometalates (POMs) are a series of compounds composed of metals (mainly formed by transition metals Mo, W, and V) and oxygen which have great catalytic performance [24]. Many researchers have introduced Keggin-type POMs into oxidative desulfurization systems [25,26]. Wang et al. immobilized molybdenum-containing polyoxometalate clusters on an organic framework using the ship-in-bottle method, and the desulfurization rate of DBT reached 98.5% within 30 min at 30 °C [27]. Zhang et al. prepared a novel vanadium-substituted polyoxometalate ionic liquid, and the results of the experiments demonstrated that the removal of DBT reached up to 98.9% under optimal conditions [28].

Polyvinylimidazole is formed by repeated covalent bonds of 1-vinylimidazole. Therefore, the polymer surface has a number of binding sites. More importantly, the nitrogen on the imidazole group can be complexed with a variety of substances [29]. The resulting catalyst has better dispersibility, which achieves higher catalytic efficiency. Xu et al. prepared microsphere ionic liquid catalysts through polyvinylimidazole, 1-chlorobutane, and cobalt chloride. The catalysts can remove more than 99% of DBT in a short time [30]. Yang et al. used vinyl imidazole with different carbon chain lengths to polymerize and self-assemble with ammonium molybdate and achieved the effect of deep desulfurization after 90 min of reaction [31].

In this work, a hypercross-linked polymer complexed heteropolyacid catalyst was designed by introducing heteropolyacids onto the surface of polyvinylimidazole. The imidazole group within polyvinylimidazole acts as a linking agent, making the complex more compact and thereby enhancing desulfurization efficiency. The structure, morphology, and desulfurization performance of the catalyst were systematically studied. Notably, the desulfurization efficiency of VE-HPW remained at 98.5% even after nine cycles of reuse, indicating its potential for practical applications.

## 2. Results and Discussion

### 2.1. Characterization of Catalyst

**Figure 1:**
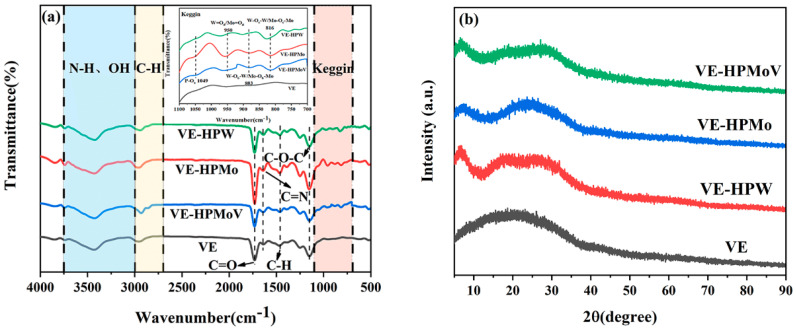
(**a**) FT-IR spectra of VE, VE-HPW, VE-HPMo, and VE-HPMoV and (**b**) XRD patterns of VE, VE-HPW, VE-HPMo, and VE-HPMoV. Different colors represent different samples.

FT-IR was applied to identify the functional groups on VE, VE-HPW, VE-HPMo, and VE-HPMoV (Figure 1a). The broad absorption peak between 3700 and 3200 cm^−1^ is attributed to the OH groups and N-H stretching vibrations. The peaks around 2960 cm^−1^ correspond to asymmetrical C-H stretching, while the peak at 2880 cm^−1^ is associated with symmetrical C-H stretching [32,33,34]. The intense peaks at 1730 and 1150 cm^−1^ are related to the C = O stretching vibration and C-O-C stretching vibration, respectively [35,36]. A low-intensity band is discovered at 1642 cm^−1^, which is attributed to the C = N vibration of the imidazole ring groups. The peak at 1461 cm^−1^ belongs to the C-H asymmetric bending vibration [37]. It is generally characterized by four vibration absorption peaks appearing from 1100 to 700 cm^−1^, illustrating the Keggin structure. These four main absorption peaks of 1049, 950, 883, and 816 cm^−1^ correspond to the characteristic stretching vibration of the chemical bonds of P-O_a_, W = O_d_ or Mo = O_d_, W-O_b_-W or Mo-O_b_-Mo, and W-O_c_-W or Mo-O_c_-Mo, respectively [38,39]. It is certified that the heteropolyacid and VE successfully reacted.

The XRD patterns of VE, VE-HPW, VE-HPMo, and VE-HPMoV were analyzed, as shown in Figure 1b. It can be observed that VE only has a broad peak around 20° in the XRD spectrum, indicating that VE has an amorphous structure [40,41]. The catalysts of VE-HPW, VE-HPMo, and VE-HPMoV also show amorphous structures. The catalysts have a series of diffraction peaks between 5° and 40°, which are caused by the diffraction of heteropolyacid anions. The peaks of the Keggin structure of the heteropolyacid almost disappear, which is mainly due to the sharp characteristic peak that is obscured by the broad peak. Compared with VE-HPMo, and VE-HPMoV, the peak width of VE-HPW in the range of 12–40° is wider, indicating that they are composed of several signals. This is also one of the important factors why VE-HPW outperforms the other two catalysts in oxidative desulfurization.

**Figure 2:**
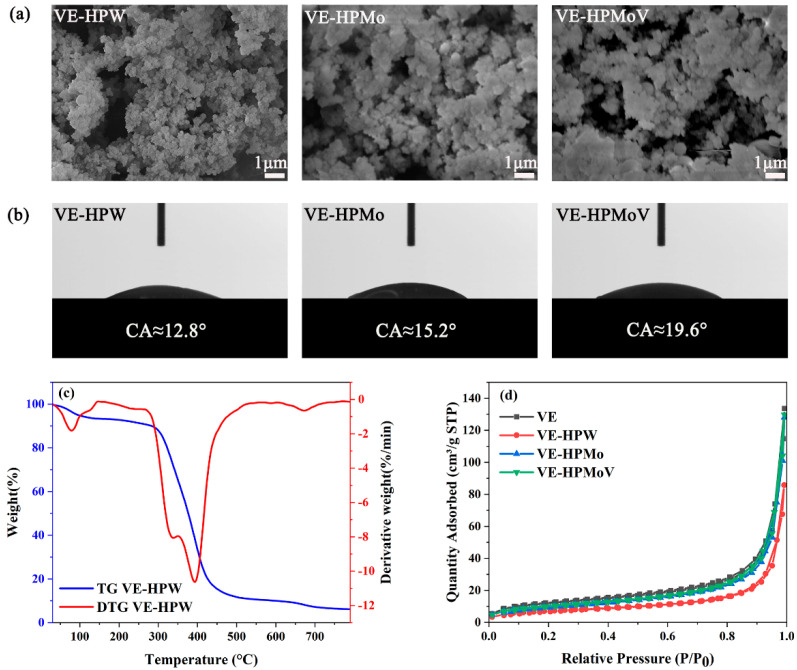
(**a**) SEM images of VE-HPW; VE-HPMo; VE-HPMoV. (**b**) Contact angle on surface of VE-HPW; VE-HPMo; VE-HPMoV. (**c**) TG and DTG curves of VE-HPW. (**d**) N_2_ adsorption–desorption isotherms.

The morphology and surface characteristics of the synthesized samples were examined using SEM (Figure 2a). VE-HPW exhibited a small, fluffy, nanoscale, coral-like structure with well-formed pores on the surface, facilitating the exposure of more active sites. In contrast, VE-HPMo and VE-HPMoV showed agglomeration, which tends to encapsulate the active sites within the structure.

We further studied the hydrophilicity of the catalysts by pressing them into a sheet. Figure 2b shows the change in the contact angle of different catalysts. When the water drops come into contact with the catalyst surface, catalysts can be instantly wetted. The contact angles of VE-HPW, VE-HPMo, and VE-HPMoV are about 12.8°, 15.2°, and 19.6°, respectively. Therefore, the catalysts are hydrophilic [42]. The smaller the contact angle, the better the wettability and hydrophilicity of the catalysts. During the desulfurization reaction, the catalysts can better contact hydrogen peroxide, thereby improving the desulfurization efficiency.

Thermogravimetric analysis (TGA) was employed to investigate the thermal stability of the catalysts within the temperature range of 30–800 °C. The results from both the TG and DTG analyses of the catalysts are presented in Figure 2c and Figures S1–S3. The first mass loss occurred below 110 °C, and the mass loss percentages of VE, VE-HPW, VE-HPMo, and VE-HPMoV are 3.2%, 5.4%, 3.5%, and 5.3%, respectively. The weight loss curve is a gentle descent due to the removal of physically adsorbed water on the catalyst surface. The mass loss in the second stage is mainly due to the disappearance of bound water in the catalysts (120–280 °C). As the temperature increased to 280 °C, the catalysts began to decompose in large quantities until 450 °C, and the mass loss of the catalysts was about 80% during this process. It can be seen from the DTG diagram that the mass change rate is the largest near 400 °C, which is caused by the decomposition of the imidazole ring [43]. Mass loss is also observed for the catalysts of VE-HPMoV and VE-HPMo above 600 °C, which may be related to the decomposition of the heteropolyacid anion (Figures S2 and S3). Above 700 °C, thermal decomposition of the catalysts was completed, and the quality gradually became stable. The final quality of the catalysts is VE-HPW > VE-HPMo > VE-HPMoV, indicating that VE-HPW has the best thermal stability. The experimental results are consistent with the observation that the performance of the catalyst in oxidative desulfurization is superior to that of the other two catalysts.

The catalysts of VE, VE-HPW, VE-HPMo, and VE-HPMoV were characterized by N_2_ adsorption–desorption, and their pore structure and specific surface area were analyzed. In Figure 2d, all of the samples indicate type II isotherm, based on the IUPAC classification that expresses the typical physical adsorption process on non-porous or macroporous adsorbents [44,45]. The catalysts exhibit an H3 hysteresis loop in which the saturated adsorption plateau is not obvious. This testifies that the pore structure of the catalysts is very irregular, which is consistent with the irregular coral-like morphology displayed in the SEM images. According to the findings presented in Table 1, the catalysts exhibit a gradual reduction in both specific surface area and pore volume following the addition of HPA. This can be attributed to the presence of HPA on the outer surface of VE, which acts as an obstruction, impeding the openings of micropores and consequently resulting in a decrease in pore volume [46]. It is worth noting that all of the catalysts analyzed in this study possess an average pore size ranging from 21 to 23 nm, categorizing them as mesoporous catalysts.

**Figure 3:**
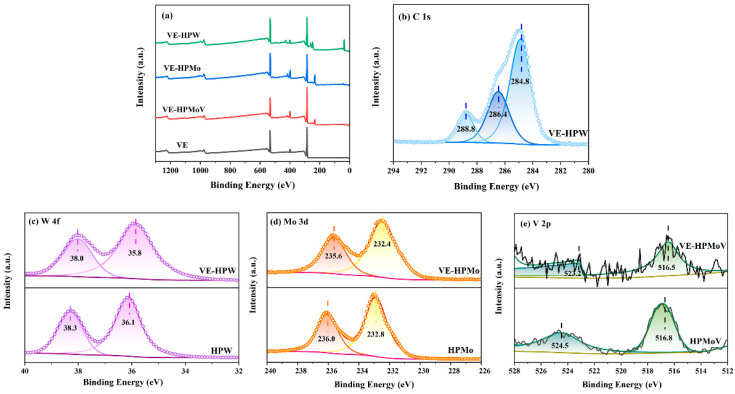
XPS spectra of (**a**) survey; (**b**) C 1s of VE-HPW; (**c**) W 4f of VE-HPW and HPW; (**d**) Mo 3d of VE-HPMo and HPMo; (**e**) V 2p of VE-HPMoV and HPMoV. Different colors represent chemical states of the catalysts.

The elemental composition and chemical state of the catalysts were further investigated using XPS. The fully scanned spectrum of the catalysts, shown in Figure 3a, confirms the presence of C, N, and O in VE. In addition to the C 1s, N 1s, and O 1s signals, the appearance of P 2p, Mo 3d, V 2p, and W 4f signals in the VE-HPA composite further verifies the successful synthesis of polyvinylimidazole and heteropolyacid.

The C 1s spectrum (Figure 3b) can be divided into three peaks at 284.8, 286.4, and 288.8 eV, corresponding to the C-C, C-O-C, and O-C = O of VE-HPW. Comparing the XPS of HPA and VE-HPA, the peaks of the Mo, V, and W elements all shift to the lower binding energy direction, which illustrates that the electrons are transferred in the process of catalyst preparation.

We treated the metal elements in the catalysts separately (Figure 3c–e). It can be seen in Figure 3c that the W 4f orbital in HPW consists of two peaks of W 4f_5/2_ and W 4f_7/2_ at 38.3 eV and 36.1 eV, respectively (the spin–orbit component is 2.2 eV). The two peaks of 38.0 eV and 35.8 eV correspond to the W 4f_5/2_ and W 4f_7/2_ spin states, respectively, demonstrating the strong bonding between VE and HPW [46,47]. The W element of the catalysts HPW and VE-HPW exists in the form of a positive hexavalent, indicating that the valence of tungsten does not change during the reaction.

The fitting of Mo elements shows that all Mo elements have only one pair of fitted peaks, and the interval between the two peaks is 3.2 eV (Figure 3d). Taking VE-HPMo as an example, the two peaks at the binding energies of 235.6 eV and 232.4 eV are assigned to Mo 3d_5/2_ and Mo 3d_3/2_, respectively. It is revealed that Mo exists in the form of +6 valence in the catalyst VE-HPMo [48]. Moreover, the Mo element exists in the form of +6 valence in other catalysts. This shows that the valence of molybdenum did not change during the reaction.

As depicted in Figure 3e, the V 2p_1/2_ and V 2p_3/2_ binding energies of pure HPMoV are located at 524.5 and 516.8 eV, respectively. The peak intensity of V 2p_3/2_ is higher than that of V 2p_1/2_, which indicates that the valence state of the V element is V^5+^. When VE is connected, the binding energy of VE-HPMoV moves to the lower energy level, and there is electron transfer between VE and HPMoV.

### 2.2. Catalytic Performance

**Figure 4:**
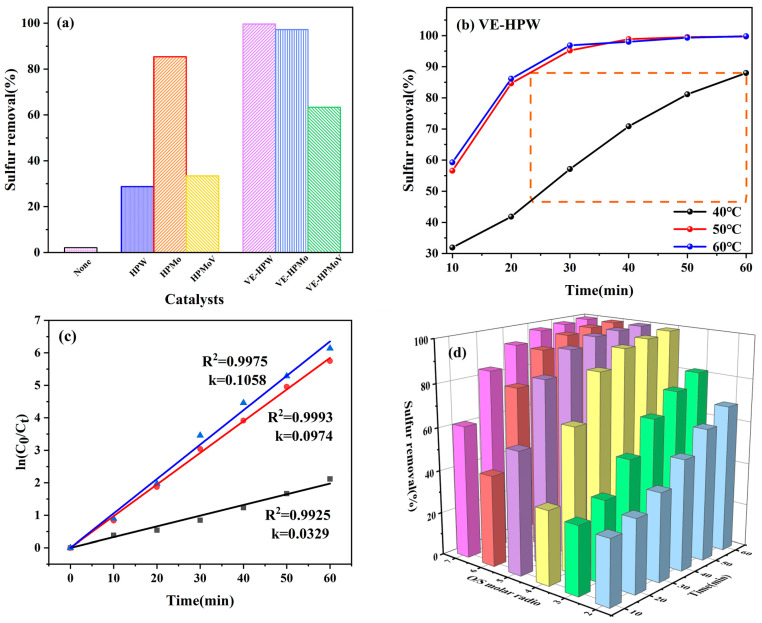
(**a**) The effects of the different catalysts on the conversion of DBT by ODS. Reaction conditions: catalyst = 0.05 g, T = 50 °C, and O/S = 5, t = 60 min. (**b**) The effects of the reaction temperature on the conversion of DBT by ODS. Reaction conditions: catalyst (VE-HPW) = 0.05 g, O/S = 5, and t = 60 min. (**c**) Pseudo-first-order kinetics for the oxidation of DBT under different reaction temperatures in the ODS system. Experimental conditions: m (catalyst) = 0.05 g, O/S = 5, and V (model oil) = 5 mL. (**d**) The effects of O/S on the conversion of DBT by ODS. Reaction conditions: catalyst (VE-HPW) = 0.05 g, T = 50 °C, and t = 60 min. Different colors represent different catalysts.

To investigate the impact of various reaction conditions on desulfurization efficiency, the oxidative desulfurization activities of the catalysts are illustrated in Figure 4. Without catalysts, the desulfurization efficiency of H_2_O_2_ is only 2.1%. In comparison to HPW, HPMo, and HPMoV, the desulfurization rates of VE-HPW, VE-HPMo, and VE-HPMoV are significantly improved (Figure 4a). Under the same conditions, VE-HPW achieves 97.7% removal of DBT at 50 °C within 60 min.

As the reaction temperature was elevated from 40 °C to 50 °C, there was a noteworthy enhancement in the efficiency of desulfurization, with the values rising from 87.96% to an impressive 99.68%. The maximum desulfurization efficiency of the VE-HPW catalyst at 40 °C equals the desulfurization efficiency in the 23rd minute at 50 °C. Compared with 50 °C, the desulfurization efficiency was virtually unchanged at 60 °C (Figure 4b). The removal rate of DBT at 60 °C is lower than at 50 °C when VE-HPMoV is used as the catalyst. The reason is that H_2_O_2_ in the system accelerates decomposition after the temperature rises, reducing the oxygen molecule utilization rate (Figure S4). Therefore, 50 °C is chosen as the efficient temperature.

The pseudo-first-order kinetic diagram of the VE-HPW catalyst at different temperatures is shown in Figure 4c. Obviously, the rate constant k = 0.1058 min^−1^ is better than other conditions. The activation energy (Ea) calculated by the Arrhenius equation is 39.01 kJ mol^−1^.

The amount of H_2_O_2_ has an important influence on oxidative desulfurization performance. It is shown in Figure 4d that when O/S = 5, the desulfurization effect of VE-HPW is the best. When the amount of H_2_O_2_ is too much, the water produced will reduce the catalytic activity, thereby inhibiting the desulfurization process (Figure S5). In a comprehensive analysis, O/S = 5 is the optimal oxygen–sulfur ratio.

**Figure 5:**
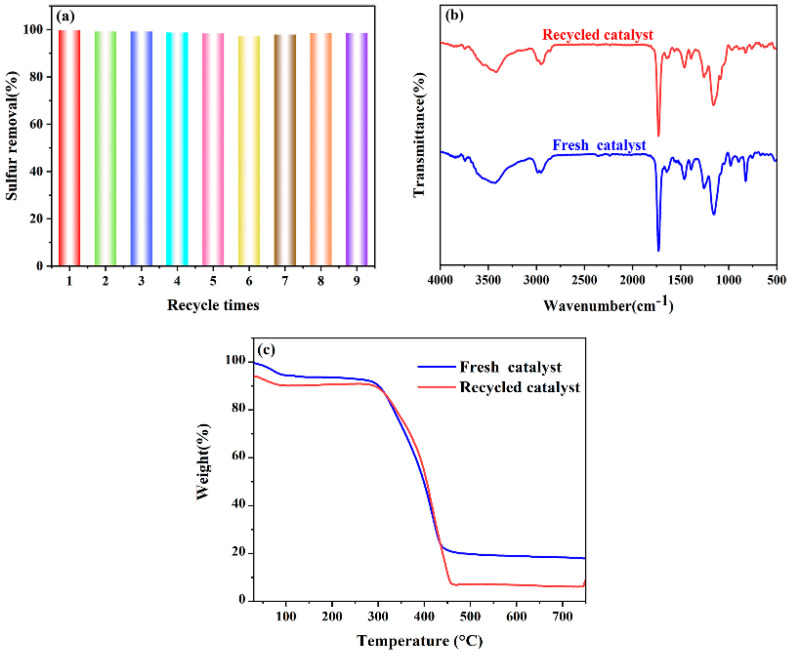
(**a**) The recycling performance of the catalyst. Reaction conditions: catalyst (VE-HPW) = 0.05 g, T = 50 °C, O/S = 5, and t = 60 min. (**b**) FT-IR of fresh and recycled catalyst. (**c**) TG analysis of fresh and recycled catalyst.

The cycle performance is an important criterion for investigating the pros and cons of the catalysts. It also determines the prospect of the catalysts in industrial applications. VE-HPW is taken as an example to further test its reuse efficiency. The upper layer of clarified oil in the reaction flask was removed after every ODS reaction. The reaction flask was dried in a drying oven at 65 °C for 24 h to obtain the catalyst, which was then reused in subsequent catalytic cycles under the same reaction conditions. As shown in Figure 5a, the catalyst still achieved a sulfur removal capacity of 98.60% after the ninth recycling. This indicates that the catalyst maintains a stable structure under the conditions studied, demonstrating satisfactory reusability in oxidative desulfurization processes.

The catalyst before and after the reaction was characterized by FT-IR (Figure 5b) and the peak in the figure has no significant change. This shows that the composition and structure of the catalyst after the reaction have not changed, which further proves that the catalyst prepared using the cross-linking method has extraordinary stability [49].

The catalyst before and after the reaction was characterized by TG analysis (Figure 5c). The general trend of the catalyst’s thermal stability curve before and after the reaction is the same. This proves the good stability of the catalyst VE-HPW in themodel oil.

### 2.3. Proposed Mechanism

**Figure 6:**
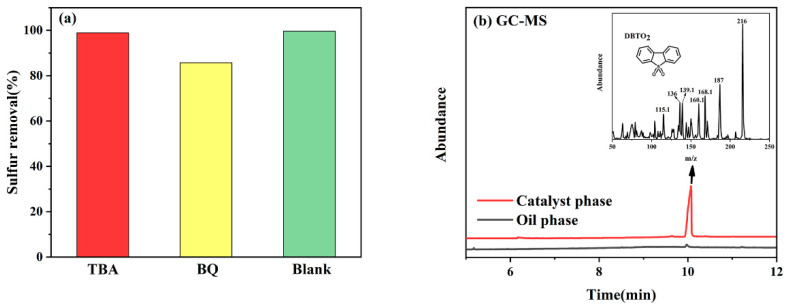
(**a**) Investigation of selective quenching experiments and (**b**) GC–MS analysis of oil phase after reaction.

To further explore the types of free radicals generated during the ODS process of VE-HPW, p-benzoquinone (BQ) and tert-butanol (TBA) were chosen as the trapping agents for O_2_^−^ and OH in the reaction system, respectively. The process of the free radical capture experiment is similar to the ODS process, adding 50 mg of the catalyst, 5 mL of the model oil, and 1 mL of ionic liquid, with constant stirring. Then, BQ or TBA and a certain volume of H_2_O_2_ are added, and timing begins. After the reaction is completed, the remaining sulfur compound content in the simulated oil is detected by GC. Figure 6a shows that the removal rate of DBT decreased after adding BQ and TBA, but the capture effect of tert-butanol was more obvious. It can be inferred that both OH and O_2_^−^ were generated [50].

After desulfurization, the oil phase can be directly filtered through a filter membrane and then used for GC-MS detection (Figure 6b). The catalyst phase, however, first requires the removal of any residual simulated oil on its surface. It is then extracted with acetonitrile to assess the recycling performance of the catalyst. A higher peak was detected at 10.0 min, which is the peak of DBTO_2_ (m/z = 216). No peak was found at the time of 7.4 min, so there was no DBT in either the oil phase or the catalyst phase [51,52], indicating that DBT was completely converted into DBTO_2_ during the reaction.

**Scheme 1:**
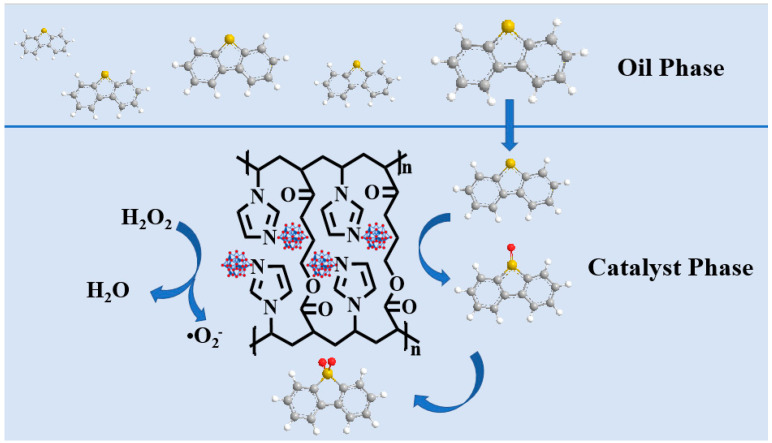
The proposed mechanism for the oxidation of DBT. Different colors represent different elements.

Through the experimental characterization results of the free radical trapping experiments and GC-MS, it was verified that the oxidation product was DBTO_2_. The reaction mechanism of the ODS system was speculated and is shown in Scheme 1. Firstly, DBT was adsorbed on the surface of the catalyst VE-HPW and then formed a DBT radical (DBT^+^) by reacting with the active center in the catalyst. When oxidized by H_2_O_2_, superoxide radicals (O_2_^−^) are generated. Secondly, DBT was oxidized to DBTO. Finally, further oxidation generated the final oxidation product DBTO_2_.

## 3. Experimental Section

### 3.1. Materials

1-Vinylimidazole (99%), phosphotungstic acid hydrate (AR), and tetradecane (AR, 99%) were received from Aladdin, China. Ethylene glycol dimethacrylate (98%), acetonitrile (≥99.9%), phomolybdic acid hydrate (AR), vanadium (V) oxide (99.99%), molybdenum trioxide (AR, 99.5%), phosphoric acid (AR, purity ≥ 85 wt.% in H_2_O), 1-methyl-3-octylimidazolium tetrafluoroborate (Moim^+^BF_4_^−^) (98%), dibenzothiophene (DBT, 99%), octane (>99%), and *p*-benzoquinone (≥99.5%) were all purchased from Shanghai Macleans Biochemical Technology Co., Ltd. Ethanol (≥99.7%) and hydrogen peroxide (30 wt%) stemmed from Xilong Chemical Co., Ltd., Tianjin, China 2,2′-Azobis(2-methylpropionitrile) was supplied by Tianjin Guangfu Fine Chemical Research Institute. Tert-butanol was obtained from Fuchen Chemical Reagent Co., Ltd., Tianjin, China. All chemical reagents were used directly without further purification.

### 3.2. Characterization

FTIR spectra of the oxide and sulfide catalysts were obtained using a Nicolet 6700 intelligent Fourier infrared spectrometer (KBr pellets) with a range of 400–4000 cm^−1^. The surface morphology of the samples was measured using a JSM-7100F field emission scanning electron microscope (SEM). X-ray powder diffraction (XRD) was performed with Ultima-IV, and the scanning rate was 2° min^−1^ in the 2θ range from 5° to 90°. X-ray photoelectron spectroscopy (XPS) data were recorded with an electron spectrometer (Thermo Scientific K-alpha, Waltham, MA, USA). The Al Kα ray (hν = 1486.6 eV) as the excitation source was used to quantitatively analyze elements and determine the element valence in the material. The binding energy of all samples was revised using the carbon C 1s peak (284.8 eV) as a reference. The catalysts were characterized by using an automatic specific surface and porosity analyzer BET (Micromerics ASAP 2460). Thermogravimetric analysis (TG) was performed using the STA 449 F3 Jupiter synchronous thermal analyzer. The temperature was 30–800 °C and the heating rate was 15 °C·min^−1^.

The sulfide concentration and products in the model oil were analyzed by gas chromatography (GC; Agilent 7890; temperature program: 100 °C; temperature rising rate: 15 °C min^−1^; 250 °C for 10 min; HP-5 MS column, 30 m × 250 μm i.d. × 0.25 μm) and gas chromatography–mass spectrometry (GC-MS; Agilent 7890–595; temperature program: 100 °C; heating rate: 15 °C min^−1^; 230 °C incubation for 10.2 min; HP-5 MS column, 30 m × 250 μm i.d. × 0.25 μm).

Contact angle measurements were performed for different catalyst layer samples. The samples were at least 3 cm × 3 cm to avoid significant changes in the samples’ surface caused by the swelling effect. The contact angle was measured using the optical contact angle measurement system OCA 20 from DataPhysics Instruments GmbH. For each measurement, a 15 μL deionized water droplet was made by placing the tip of the syringe close to the sample surface. The water droplet then attached to the sample surface. Before the water droplet attached to the sample surface, the wetting process was recorded until no further significant change at the surface was observed.

### 3.3. Synthesis of the Catalysts

#### 3.3.1. Preparation of Phosphorus Molybdenum Vanadate Heteropolyacid

Molybdenum trioxide (MoO_3_) (14.4 g) and vanadium pentoxide (V_2_O_5_) (0.82 g) were added to 250 mL of deionized water. When the reactant underwent condensation reflux, 1.15 g of 85 wt.% phosphoric acid was dropwise added and with constant stirring for 24 h. After drying for 24 h at 60 °C, the obtained product was an orange solid H_5_PMo_10_V_2_O_40_, designated as HPMoV [32].

#### 3.3.2. Synthesis of Polyvinylimidazole

The synthesis of polyvinylimidazole was based on a methodology previously published, with minor modifications [33]. 1-vinylimidazole (2 mmol), ethylene glycol dimethacrylate (10 mmol), and 2,2′-azobis(2-methylpropionitrile) (AIBN) (2 g) were dissolved in acetonitrile (20 mL) and placed in a round-bottomed flask, which was then sealed. The mixture was then purged with nitrogen for 15 min. Polymerization was performed at 65 °C for 24 h under the protection of N_2_. The resulting polymers were washed with ethanol and dried at 70 °C for 24 h. A polymer as a white solid (denoted as VE) was successfully produced.

#### 3.3.3. Synthesis of VE-HPA

Phosphotungstic acid (HPW) and VE were dispersed in absolute ethanol (20 mL) and stirred at 30 °C for 24 h. The solution was filtered and washed with absolute ethanol to remove unreacted phosphotungstic acid after the reaction. It was dried in a vacuum drying oven at 60 °C for 24 h. Finally, a white solid was obtained, which was recorded as VE-HPW. The mass ratio of HPW and VE is 1:5. The synthesis processes of polyvinylimidazolyl- phosphomolybdic acid (VE-HPMo) and polyvinylimidazolyl- phosphorus molybdenum vanadate heteropolyacid (VE-HPMoV) are similar to that of VE-HPW.

#### 3.3.4. ODS Process

**Scheme 2:**
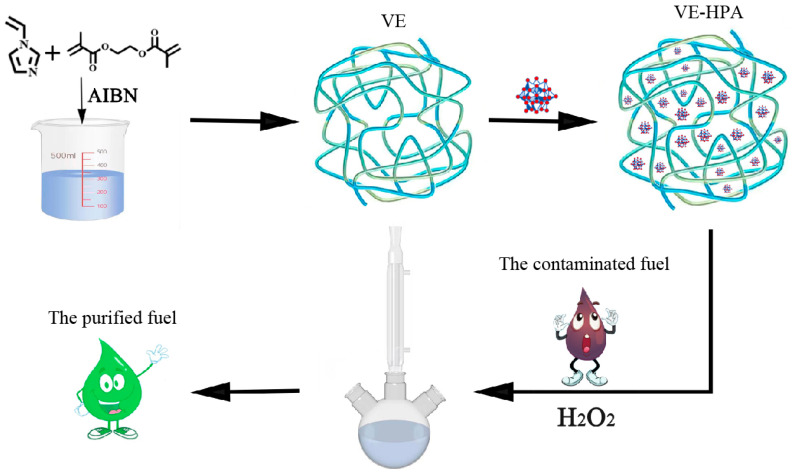
The synthesis procedure of VE-HPA and the ODS process. Different colors represent different elements.

The model oil was prepared by dissolving tetradecane and DBT in n-octane and transferring the solution to a volumetric flask. The sulfur content in the model oil is 500 ppm, and 4000 ppm of tetradecane is used as the internal standard. In the ODS process, 50 mg of each catalyst was weighed into a reactor, followed by adding 5 mL of the model oil, 1 mL of Moim^+^BF_4_^−^, and H_2_O_2_ sequentially and with constant stirring. Moim^+^BF_4_^−^ was used to extract DBT from the model oil into the ionic liquid phase, facilitating catalytic oxidation. The right amount of liquid in the octane phase was taken in the same time interval for GC detection. The synthesis procedure of VE-HPA and the ODS process are summarized in Scheme 2. The obtained model oil was tested by GC to detect the remaining sulfur content at that time. After the reaction, the residual sulfur content was tested via an internal standard (tetradecane, 4000 ppm) method using a gas chromatograph equipped with a capillary column (HP-5, 30 m 0.25 mm 0.25 μm). The injector temperature was 300 °C and the detector temperature was 250 °C. The temperature of the GC process started at 100 °C and rose to 250 °C at 15 °C/min. Sulfur removal (%) was calculated using the following equation:

(1) $$Sulfur\;removal\left(\%\right)=\frac{Initial\;sulfur\;content-Residual\;sulfur\;content}{Initial\;sulfur\;content}\times 100\%$$

#### 3.3.5. Recovery of Catalyst

After each ODS reaction, the catalyst was separated from the oil, and the oil was decanted. The entire sequence of these processes is considered a single reaction cycle. The recovered catalyst was then used in subsequent cycles, repeating the aforementioned steps until a significant decrease in catalyst activity was observed.

## 4. Conclusions

In summary, porous polymer polyvinylimidazoles were successfully created using the facile ontology aggregation method. Heteropolyacid was loaded into VE using the hydration dispersion method to form the catalyst VE-HPA. When compared with other samples, the VE-HPW catalyst, which incorporates heteropolyacid as the active center, demonstrated remarkable oxidative desulfurization activity. As a result, it emerged as the leading catalyst among the group, showcasing its superiority. The results proved that 99.68% of DBT can be removed using VE-HPW in 60 min at 50 °C. After nine cycles, there is no significant decrease in the catalytic efficiency. This shows that the catalyst VE-HPW has a bright future in practical applications.

## Figures and Tables

**Table 1:** Physical property parameters of catalysts VE, VE-HPW, VE-HPMo, and VE-HPMoV.

Entry	Samples	Specific Surface Area (m^2^/g)	Pore Volume (cm^3^/g)	Pore Size (nm)
1	VE	43.67	0.087	21.33
2	VE-HPW	26.24	0.053	22.69
3	VE-HPMo	37.52	0.079	23.02
4	VE-HPMoV	37.86	0.084	22.84

## Data Availability

Data is contained within the article and Supplementary Material.

## Supplementary Materials

The following supporting information can be downloaded at: https://www.mdpi.com/article/10.3390/molecules29174238/s1. Figure S1. TG and DTG curves of VE. Figure S2. TG and DTG curves of VE-HPMoV. Figure S3. TG and DTG curves of VE-HPMo. Figure S4. Effects of the reaction temperature on the conversion of DBT by ODS. Reaction conditions: Catalyst (VE-HPMoV) = 0.05 g, O/S = 5, t = 60 min. Figure S5. Effects of the O/S on the conversion of DBT by ODS. Reaction conditions: Catalyst (VE-HPMoV) = 0.05 g, T = 50 °C, t =60 min.
