# Supplementary material for: Cross-Linked Polyvinylimidazole Complexed with Heteropolyacid Clusters for Deep Oxidative Desulfurization
Source: Molecules. 2024 Sep 6;29(17):4238. doi: 10.3390/molecules29174238 (PMC11396842; doi:10.3390/molecules29174238)
Supplement: Supplementary file 1 [file molecules-29-04238-s001.zip › molecules-3061440-supplementary.pdf]

Cross-Linked Polyvinylimidazole Complexed with Heteropolyacid Clusters for Deep Oxidative Desulfurization

Zhuoyi Ren [1], Jiangfen Sheng [2], Qibin Yuan [1], Yizhen Su [1], Linhua Zhu [1,*], Chunyan Dai [1,*] and Honglei Zhao [3,*]

[1] Engineering Research Center of Tropical Marine Functional Polymer Materials of Hainan Province, Key Laboratory of Water Pollution Treatment and Resource Reuse of Hainan Province, Key Laboratory of Functional Organic Polymers of Haikou, Hainan Normal University, Haikou 571158, China
[2] Jiangsu Jitri Carbon Fiber & Composite Application Technologies Research Institute Co., Ltd., Changzhou 213000, China
[3] Hainan Lesso Technology Industrial Co., Ltd., Dingan 571200, China
* Correspondence: zhulinhua@hainnu.edu.cn (L.Z.); daichunyan@hainnu.edu.cn (C.D.); dalspgb@lesso.com (H.Z.)

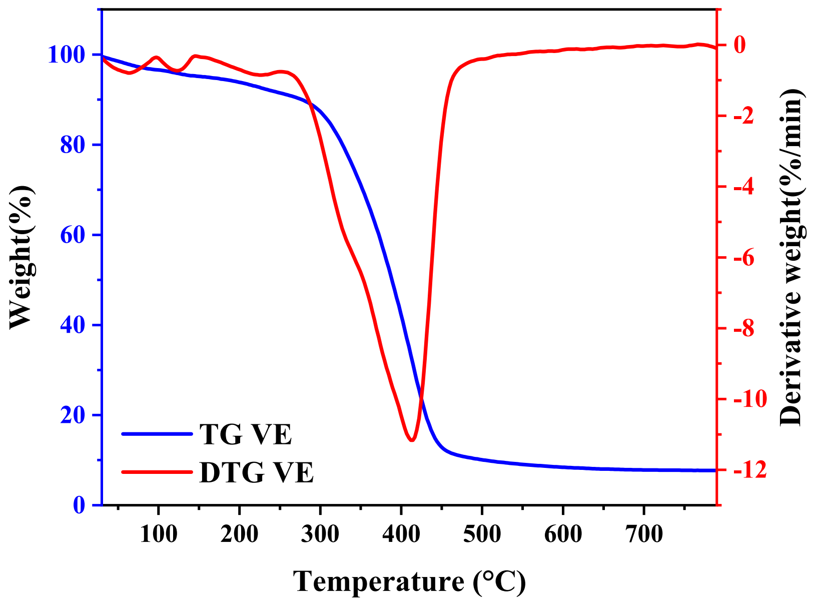

Figure S1. TG and DTG curves of VE.

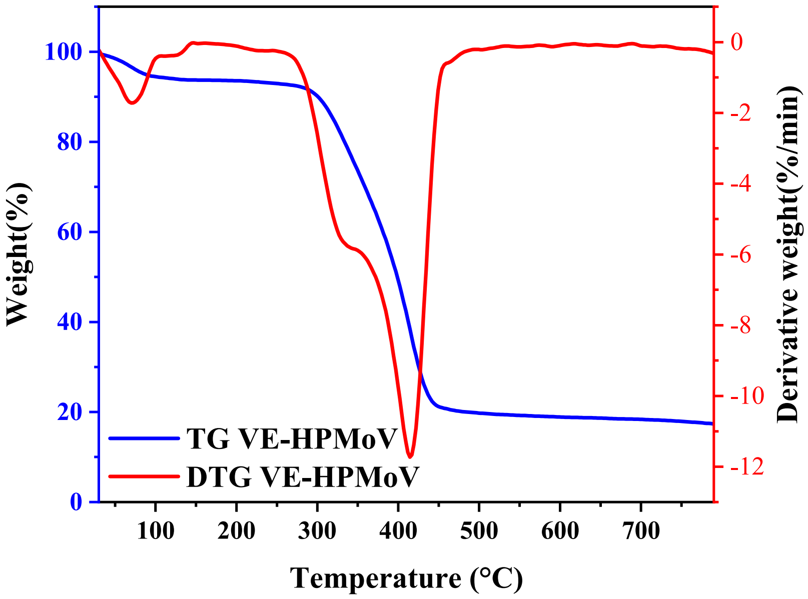

Figure S2. TG and DTG curves of VE-HPMoV.

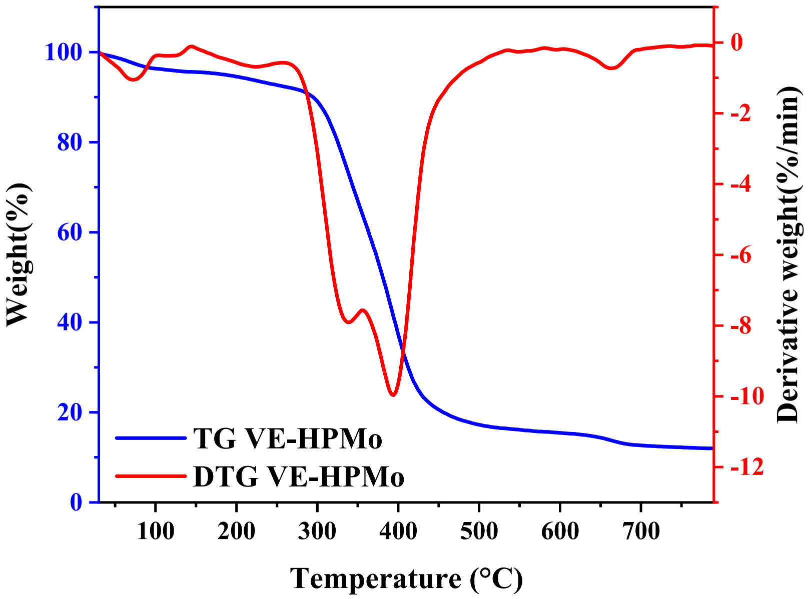

Figure S3. TG and DTG curves of VE-HPMo.

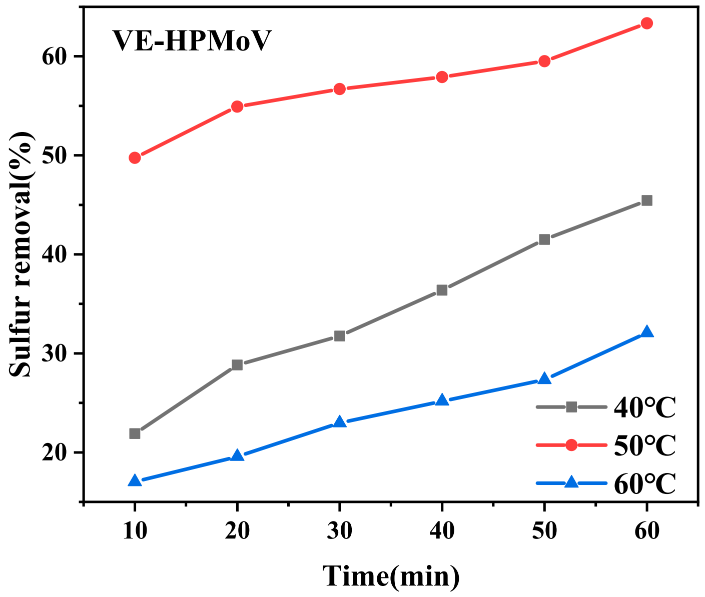

Figure S4. Effects of the reaction temperature on the conversion of DBT by ODS.

Reaction conditions: Catalyst (VE-HPMoV) = 0.05 g, O/S = 5, t = 60 min.

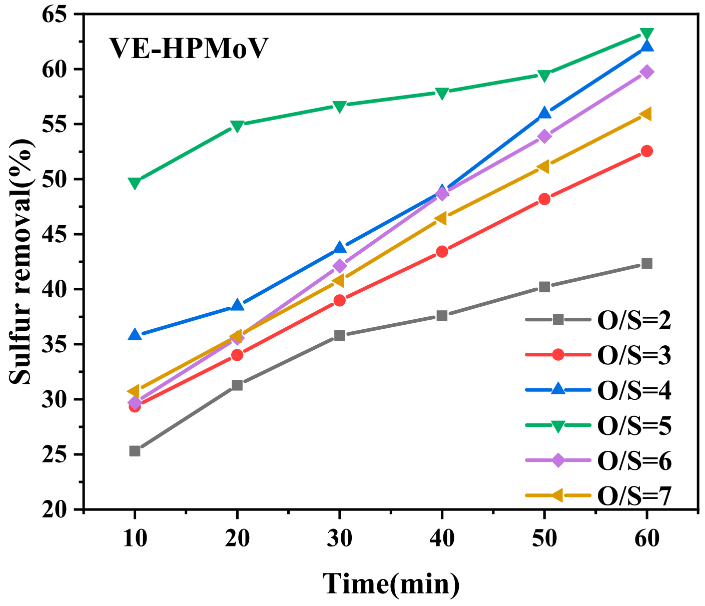

Figure S5. Effects of the O/S on the conversion of DBT by ODS.

Reaction conditions: Catalyst (VE-HPMoV) = 0.05 g, T = 50 °C, t =60 min.
